# The Spectrum of Long-Term Behavioral Disturbances and Provided Care After Traumatic Brain Injury

**DOI:** 10.3389/fneur.2020.00246

**Published:** 2020-04-07

**Authors:** Marlies L. Timmer, Bram Jacobs, Marleen C. Schonherr, Jacoba M. Spikman, Joukje van der Naalt

**Affiliations:** ^1^Department of Neurology, University of Groningen, University Medical Center Groningen, Groningen, Netherlands; ^2^Department of Rehabilitation Medicine, University of Groningen, University Medical Center Groningen, Groningen, Netherlands; ^3^Department of Clinical Neuropsychology, University of Groningen, University Medical Center Groningen, Groningen, Netherlands

**Keywords:** behavioral disturbances, traumatic brain injury, outcome, return to work, discharge destinations, caregivers

## Abstract

**Introduction:** Behavioral disturbances are found in 50–60% of traumatic brain injury (TBI) survivors with an enormous impact on daily functioning and level of recovery. However, whether typical profiles can be distinguished and how these relate to provided care is unclear. The purpose of this study is to specify the characteristics of behavioral disturbances in patients with various severity of TBI and the impact on functional outcome. Furthermore, the pathways of care after hospital discharge for patients and their care givers are analyzed.

**Methods:** We performed a retrospective cohort study comprising 226 patients with mild TBI (mTBI; *n* = 107) and moderate-to-severe TBI (mod/sevTBI; *n* = 119) treated at the outpatient clinic and/or rehabilitation center of our university hospital between 2010 and 2015. Inclusion criteria were: behavioral disturbances as determined with the Differential Outcome Scale and age ≥16 years. Functional outcome was determined by the Glasgow Outcome Scale Extended and return to work (RTW) at six months to one year post-injury. Behavioral impairments and pathway of care were derived from medical files and scored according to predefined criteria.

**Results:** Overall 24% of patients showed serious behavioral disturbances; three times higher in mod/sevTBI (35%) compared to mTBI (13%). mTBI patients mostly showed irritation (82%) and anger (49%), while mod/sevTBI patients mostly showed irritation (65%) and disinhibition (55%). Most (92%) patients returned home, half of the patients did not RTW. Deficits in judgment and decision-making increased risk of no RTW 10-fold. One in ten patients was (temporarily) admitted to a nursing home or psychiatric institution. 13% Of caregivers received support for dealing with impairments of patients and 13% of the mTBI and 17% of the mod/sevTBI patients experienced relational problems.

**Conclusions:** The spectrum of behavioral disturbances differs between TBI severity categories and serious behavioral disturbances are present in a quarter of patients. Only half of the patients resumed work regardless of severity of injury suggesting that particularly the presence and not the severity of long-term behavioral disturbances interferes with RTW. Most patients returned home despite these behavioral disturbances. These findings underline the importance of early identification and appropriate treatment of behavioral disturbances in TBI patients.

## Introduction

Traumatic brain injury (TBI) is a public health problem worldwide and an important cause of neurological and psychosocial dysfunction (1, 2). The estimated incidence of TBI in Europe is 235/100.000 cases per year (3). The severity of TBI is generally determined by using the Glasgow Coma Scale (GCS) and is characterized as mild, moderate or severe TBI. A large number of TBI survivors, especially those with moderate to severe TBI (mod/sevTBI), has permanent impairments regarding the physical, cognitive and behavioral domains and social functioning (4–6). Behavioral disturbances interfere with daily life and social interaction and vary from apathy, disinhibition, and agitation, to aggression and violent behavior, that frequently exist simultaneously (7, 8). These behavioral disturbances are in general difficult to manage due to impaired self-awareness and may still be present several years after trauma (9–11). In severe TBI behavioral disturbances have been found in 50–60% of survivors with an enormous impact on participation, in particular vocational and family functioning (12–16) but limited information is available on the presence and effect of behavioral disturbances in mTBI patients. It is known that frontal CT-abnormalities are associated with long-term neurobehavioral changes in mod/sevTBI (17) and that lesions in the prefrontal and temporal cortex are associated with aggression, violence, and apathy (18, 19). In mild TBI (mTBI) however, not the location of the lesion but duration of post traumatic amnesia is the most consistent predictor for behavioral impairments post-trauma (20).

After discharge from the hospital, different pathways of care emerge for patients with TBI, but it is unclear how healthcare is provided specifically for TBI patients with behavioral disturbances. The usual rehabilitation pathway for TBI patients is not suitable for some patients due to serious behavioral disturbances and impaired self-awareness and they are therefore either discharged prematurely to their homes or admitted to a nursing home or psychiatric institution (21). It has been shown that several years post-trauma 10% of TBI patients still visit a psychiatrist (22). Behavioral disturbances in TBI patients also affect the lives of their caregivers and significant others (23, 24). In a previous study half of these caregivers reported elevated distress with the severity of injury associated with family burden (15). Therefore, when evaluating long term behavioral disturbances the effect on caregivers has to be taken into account simultaneously (13).

The purpose of the current study is to identify common characteristics of behavioral disturbances in patients with TBI of various severities and to determine the association with long term outcome and return to work. A second aim is to investigate the pathways of care provided for TBI patients with behavioral disturbances, in order to evaluate which care is provided and whether this relates to outcome. Furthermore, we want to identify the impact of behavioral disturbances on their caregivers and significant others, while this is an important aspect of outcome that is not assessed with the frequently used questionnaires determining functional outcome, like the Glasgow Outcome Scale.

## Methods

### Participants

All adult TBI patients (aged ≥16 years) with post-traumatic behavioral disturbances who were treated between 2010 and 2015 at the University Medical Center Groningen (UMCG) or the UMCG Center for Rehabilitation Beatrixoord, were included if the injury had occurred in 2005 or later. Patients with disturbances based on their score on the behavorial domain of the Differential Outcome Scale (DOS) (explained below), as registered in the prospective Neurotrauma Database of our department, were included. The following demographic and clinical variables were used for analysis: age and gender, medical history with psychological or psychiatric disorder(s), substance abuse and previous TBI, severity of TBI (duration of loss of consciousness (LOC)/posttraumatic amnesia (PTA), initial Glasgow Coma Scale (GCS) score, admission to ICU, total duration of admission to the hospital).

Based on the GCS score (25), two TBI severity groups were defined: 1. Mild TBI (GCS 13-15; mTBI) and 2. Combination of moderate (GCS 9-12; modTBI) to severe (GCS ≤ 8; sevTBI) TBI. Structural traumatic brain damage was evaluated by CT and/or MRI scanning performed directly after trauma or during follow-up. The location of lesions was scored as frontal, temporal, fronto-temporal, and parieto-occipital. Information on caregiver burden and/or support and relationship problems was derived from the medical charts. This study was performed in compliance with the ethical regulations of our institute.

### Measures

Time of assessment varied depending on severity of injury: for mTBI this was in general after six months and in mod/sevTBI this was in general one year post-injury. In general for mild TBI the outcome endpoint is reached at six months post-injury and for mod/sevTBI this is at one year after trauma. (26, 27).

#### Severity of Behavioral Disturbances

Differential Outcome Scale (DOS) (28): The DOS scale categorizes outcome in four domains: neurophysical, cognitive, behavioral, and social. The DOS behavioral subscale (DOS-BS) has five categories, ranging from 5 = complete recovery or minor changes, 4 = mild changes, noted by experts or by those who knew the patient before the injury, 3 = obvious changes, noted by laymen who did not know the patient before the injury, 2 = severe personality changes and 1 = persistent vegetative state. Patients with a DOS-BS score between 2-4 were included in the study. DOS scores were obtained at the out-patient clinic of the Neurology Department of our hospital during follow-up within one year after injury. For logistic regression analysis, a dichotomy was used: mild behavioral disturbances (DOS-BS = 4) and serious behavioral disturbances (DOS-BS = 2–3).

#### Characteristics of Behavioral Disturbances

To identify behavioral disturbances data from the medical files were used, including neuropsychological examinations, reports of out-patient visits and admission reports of rehabilitation physicians and neurologists. Behavioral disturbances were scored on the following characteristics of behavior: inhibition, wandering behavior, different aspects of anger (with increasing severity order: irritation/agitation, anger, verbal aggression and (physical) violent behavior), apathy and/or less responsive affectionate behavior. Furthermore, the presence of impaired self-awareness, deficits in judgment and decision making and planning, and regulation disorders were noted. The different characteristics of behavioral disturbances were scored as present when they were as such described in the medical file notes from out-patient visits and/or reports of neuropsychological examinations, which could have been recorded at any time during the whole period of follow-up till the final outcome measurement.

#### Functional Outcome

Glasgow Outcome Scale Extended (GOSE) (29): The GOSE is an eight point scale to determine the overall functional outcome with 8 = upper good recovery, 7 = lower good recovery, 6 = upper moderate disability, 5 = lower moderate disability, 4 = upper severe disability, 3 = lower severe disability, 2 = vegetative state and 1 = death. For statistical reasons we dichotomized functional outcome in favorable versus unfavorable outcome. To compare mTBI and mod/sevTBI properly, we chose one cut-off point: favorable outcome was defined as GOSE 5-8 and unfavorable as GOSE 1-4 (30). Normally, in mTBI a favorable outcome is defined as GOSE 7-8.

#### Return to Work

Return to work (RTW) (31) was classified into seven categories: 0 = previous job or study resumed, 1 = previous job or work resumed, but with lower requirements or part-time, 2 = simplified job or study at significant lower level, 3 = not working – no study/declared unfit, 4 = not working, nursing home/mental institution, 5 = not to judge, rehabilitation program and 6 = retired. Work resumption was defined by RTW 0-1 and incomplete work resumption was defined by RTW 2-5. Pre-injury retired or incapacitated patients were not included in the RTW-analysis.

### Evaluation of Pathways of Care and Out-Patient Follow-Up

**Figure 1:**
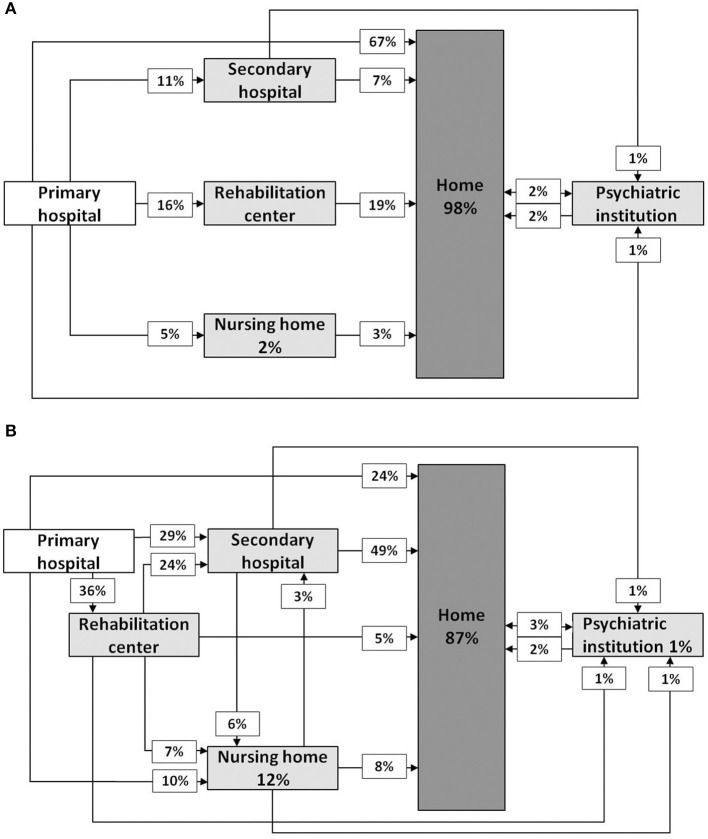
Schedule representing pathways of care after mild TBI **(A)** and moderate to severe TBI **(B)** in case of behavioral disturbances. Percentages are rounded to whole numbers.

Information on the care provided for patients was derived from medical files as well. The “primary hospital” was defined as the hospital to which the TBI patients were admitted directly after injury at the emergency department. Discharge destinations from the primary hospital or so called next level(s) of care were scored into five categories: home, regional general hospital—called “secondary hospital” in Figure 1, rehabilitation center, nursing home and psychiatric institution. The final professional care provider was defined by the last physician or therapist, next to the general practitioner, that treated the patient with persistent complaints in the chronic phase: psychiatrist, (neuro)psychologist, social worker, specialized nurse, neurologist, rehabilitation specialist or other care providers. The effect of behavioral disturbances on caregivers was measured in two ways: we registered the professional care provided for the experienced burden by care givers and whether relations of patients with their significant others had changed. As caregivers were regarded those persons that were directly responsible for the care of patients i.e., spouses, parents or children.

### Statistical Analysis

For statistical analyses Statistical Package for the Social Sciences (SPSS) version 23.0 was used. Pearson's Chi-square and Fisher's exact tests were performed for frequency analysis and unpaired t-tests were performed for differences between continuous variables (*p*-value < 0.05). Correlations between different outcome variables were calculated by Pearson correlation coefficients. Univariate binary logistic regression analysis was used to identify demographic, clinical (including behavioral characteristics) and radiological variables (mentioned above) associated with outcome parameters defined by dichotomized GOSE, DOS-BS and RTW scores. Significant variables (*p*-value < 0.05) from the univariate analysis were analyzed in multivariate logistic regression analysis with forward likelihood ratio selection, for GOSE, DOS-BS and RTW respectively. These analyses were performed for each of the severity subgroups (mTBI vs. mod/sevTBI) separately.

## Results

In total 226 patients with behavioral disorders after TBI were included in this study: 107 patients with mild TBI and 119 patients with moderate (*n* = 45) to severe (*n* = 74) TBI. Patient characteristics are presented in Table 1.

**Table 1:** Patient characteristics.

**Patients characteristics**	**mTBI *n* = 107**	**mod/sevTBI *n* = 119**	***p*-value**
Male/female ratio	71/29	72/28	0.836
Mean age at injury (SD)	45 (17)	43 (18)	0.346
Range	17-88	16-81	
Pre-injury mental problems (%)	7.5	8.4	0.797
Mechanism of injury (%)			0.121
Traffic accident	34	48	
Fall	52	45	
Violence	4.0	2.0	
Other	10	5.0	
Hospital admission (in days)			0.000
Mean (SD)	10 (12)	31 (21)	
Range	0-51	1-147	
IC admission (in days)			0.000
Mean (SD)	2.1 (5.8)	12 (11)	
Range	0-33	0-46	
CT and/or MRI lesions (%)			
None	43	20	0.000
Frontal	44	61	0.018
Temporal	27	50	0.001
Fronto-temporal	4.7	6.7	0.532
Parietal-occipital	13	14	0.839
Missing	2.8	0.8	

*mTBI, mild traumatic brain injury; mod/sevTBI, moderate to severe traumatic brain injury; SD, standard deviation; IC, intensive care; CT, computed tomography; MRI, magnetic resonance imaging*.

Overall 24% of patients showed serious behavioral disturbances (DOS-BS 2-3) and 76% showed mild behavioral disturbances (DOS-BS 4), with serious behavioral disturbances almost three times more present in severe TBI (35%) compared to mTBI patients (13%) (Table 2). Irritation and agitation were the most prevalent behavioral disturbances in mTBI (82%) and half of the patients additionally showed anger (Table 3). Most patients with mod/sevTBI were also irritated or agitated (65%), and more than half of the patients showed disinhibited behavior (55%). We found no significant gender differences in the presence of the different behavorial disturbances, only the difference in anger was significant occurring in 34% (21/61) of females and in 49% (79/160) males (Chi-square 3.984, *p* = 0.046).

**Table 2:** Outcome scores.

	**mTBI**		**mod/sevTBI**		**p-value**
	**%**	***n***	**%**	***n***	
Outcome GOSE					0.000
Favorable (5-8)	96	103	76	91	
Unfavorable (1-4)	4.0	4	24	28	
DOS behavior					0.001
Mild (4)	87	93	65	78	
Serious (2-3)	13	14	35	41	
Return to work					0.055
Yes (0–1)	53	57	51	61	
Low level/ not (2-5)	32	34	36	43	
Retired	12	13	11	13	
Missing	2.8	3	1.7	2	

*mTBI, mild traumatic brain injury; mod/sevTBI, moderate to severe traumatic brain injury; GOSE, Glasgow Outcome Scale Extended; DOS, Differentiated Outcome Scale*.

**Table 3:** Characteristics of behavioral disturbances.

Behavioral disturbances^*^	**mTBI (%)**	**mod/sevTBI (%)**	***p*-value**
Disinhibition	33	55	0.001
Wandering behavior	3.7	7.6	0.218
Different aspects of anger^*^	82	65	0.003
Irritation/agitation	82	65	0.003
Anger	49	40	0.224
Verbal aggression	11	10	0.796
Physically violent	1.9	0.8	0.504
Apathy	26	35	0.164
Less responsive affectionate behavior	3.7	3.4	0.878
Impaired self-awareness	11	30	0.000
Deficits in judgment and decision making	12	22	0.038
Planning and regulation disorder	36	41	0.256
Loss of decorum	5.6%	17%	0.002

* *Not mutually exclusive*.

*mTBI, mild traumatic brain injury; mod/sevTBI, moderate to severe traumatic brain injury*.

### Overall (Functional) Outcome

Favorable outcome (GOSE 5-8) was present in 96% of mTBI and in 24% of mod/sevTBI patients. Only about a half of all patients in both groups was able to return to previous work or study completely or part-time, with no significant difference between severity groups. No significant differences were present for GOSE scores and RTW between males and females (favorable outcome in 25 and 17%, Chi-square 1.478, *p* = 0.289) and RTW in 62 and 57% respectively (Chi-square 0.301, *p* = 0.625).

### Associations With Outcome Variables

Significant correlations were present between presence of behavioral disturbances (DOS-BS) the overall GOSE outcome score (*r* = 0.22; *p* < 0.01) and RTW (*r* = 0.41; *p* < 0.01) in mTBI. Even stronger correlations were found in mod/sevTBI between the DOS-BS and the GOSE outcome score (*r* = 0.52; *p* < 0.01) and RTW (*r* = 0.55; *p* < 0.01).

Univariate regression analyses did not show associations between the localization of brain lesions and GOSE, RTW, or DOS-BS. Variables that were significantly associated with the outcome variables (DOS-BS, GOSE, RTW) after univariate analysis (data not shown) were included in a multivariate logistic regression analysis. The individual behavioral characteristics were not analyzed with DOS-BS as dependent variable, because of obvious overlap. In mTBI multivariate analysis showed associations between DOS-BS as dependent variable and pre-injury mental health problems and substance abuse (Table 4). GOSE as dependent variable was significantly associated with age. RTW was significantly associated with the behavioral characteristic of deficits in judgment and decision-making.

**Table 4:** Multivariate logistic regression analysis.

	**Dependent variable**	**Independent variable**	**OR**	**95% C.I**.	**p-value**
mTBI	DOS-BS	Substance abuse in history	11.8	1.61;85.7	0.015
		Psychological/ psychiatric history	7.06	1.09;45.7	0.040
	GOSE	Age (years)	1.26	1.04;1.51	0.016
	RTW	Deficits in judgment and decision making	10.1	2.03;50.4	0.005
mod/ sevTBI	DOS-BS	Duration hospital admission (days)	1.03	1.00;1.05	0.010
	GOSE	Duration hospital admission (days)	1.10	1.02;1.18	0.010
	RTW	PTA duration (in days)	1.08	1.03;1.14	0.001
		Deficits in judgment and decision making	12.3	1.00;153	0.050

*mTBI, mild traumatic brain injury; mod/sevTBI, moderate to severe traumatic brain injury; DOS-BS, Differentiated Outcome Scale – Behavioral Subscale; GOSE, Glasgow Outcome Scale Extended; OR, odds ratio; C.I., confidence interval*.

In mod/sevTBI DOS-BS and GOSE were significantly associated with the total duration of hospital admission. RTW was associated with PTA duration and comparable to mTBI with deficits in judgment and decision making.

### Pathways of Care and Final Care Providers

Figure 1 shows the pathways of care for patients with mTBI and for mod/sevTBI separately. Three patients were not included due to missing data. In total 117 patients were given rehabilitation therapy either during admission at a rehabilitation center or at the out-patient clinic. Patients following a rehabilitation program showed in 54% a favorable outcome compared to 47% in patients without active rehabilitation, with 60% RTW versus 61% RTW respectively.

In mTBI four patients (4%) and in mod/sevTBI six patients (5%) were eventually admitted to a psychiatric institution, from which one patient stayed permanently in a psychiatric institution and two patients suffered from a severe depression. Both for mTBI and mod/sevTBI the psychologist or psychiatrist was in one in four patients the final care provider. In the chronic phase half of the patients was treated by a psychologist and more than 10% by a psychiatrist (Table 5). No differences regarding care providers were found for patients with mild versus serious behavioral disturbances. Eventually, 92% of the patients returned home, despite serious behavioral disturbances in a substantial proportion of the patients. Patients who stayed permanently in a nursing home all had serious behavioral disturbances.

**Table 5:** Care providers for patients in the acute and chronic phase post-injury.

	**Acute phase**		**Chronic phase**	
	**%**	***n***	**%**	***n***
*Rehabilitation physician*	52	117	65	147
*Neurologist*	66	148	62	139
*Psychiatrist*	7	16	11	25
*Psychologist*	26	59	52	117
*Social worker*	7	16	11	25
*Specialized Nurse*	17	38	9	20

*The acute phase is defined as the period between 6 weeks and 6 months after injury. The chronic phase is defined as the period 6–12 months post-injury*.

### Care Givers

In mTBI almost 13% of the patients developed relational problems, and in half of these cases the relations with their significant others were ended. In mod/sevTBI the percentage of disrupted relations was 17%, from which one third was ended. A small but significant correlation was present between the presence of behavioral disturbances and occurrence of relational problems (rho=0.23 *p* < 0.01).

In both mTBI and mod/sevTBI 13% of the caregivers received support for dealing with the impairments of the patients. This support was highly variable from a psychologist to a social worker, psycho-education group, peer support and/or family counseling. Caregivers of mod/sevTBI patients mostly received support from a psychologist (3%) or a social worker (2%) and those in the mTBI group mostly from a social worker (3%), psychologist (2%) or peer group (1%).

## Discussion

The first aim of the current study was to investigate the specific characteristics of TBI patients with behavioral disturbances and their relation to outcome. In 24% of these patients the behavioral disturbances were serious and in moderate to severe TBI serious behavioral disturbances occurred three times more than in mild TBI. A different spectrum of behavioral disturbances was found in mTBI compared to mod/sevTBI. These disturbances had a large impact on functional outcome and social life: regardless of severity of injury, half of all patients could not resume their work. Furthermore, behavioral disturbances resulted in relational problems and the termination of relationships regardless of severity of TBI. One in ten of the caregivers received support for dealing with the limitations of the patients.

One in four patients showed severe behavioral disturbances. Most mTBI patients suffered from irritability (82%), anger (49%), and disinhibition (33%) as most prominent characteristics. Moderate-to-severe TBI patients experienced significantly more disinhibition (55%), and significantly less irritation and/or agitation (65%) than patients with mTBI. The prevalence of different types of behavioral disturbances we found, are in line with findings of an earlier study (32). Others however, reported more irritability in severe TBI than in mTBI (7), but summarized only presence of behavioral disturbances without making a distinction between several subtypes of behavior as we did in the current study. A previous study showed that 69% of patients with mTBI resumed work and 44% of mod/sevTBI patients (33). Interestingly in the current study a lower percentage of RTW for mTBI was found, suggesting that the mere presence of behavioral disturbances interferes with RTW and not the severity of these behavioral disturbances. In particular the presence of deficits in judgment and decision-making increased the risk of not resuming work ten-fold. In contrast to RTW, behavioral disturbances did not have a large impact on functional outcome, since in mTBI 96%and in mod/sev TBI 76% showed an favorable outcome. These findings might be influenced by the ceiling effect of the GOSE in mTBI. We chose to dichotomize outcome scores to compare mild and mod/sev TBI patients while mTBI patients mostly score in the upper end with GOSE scores of 7 or 8. Next to this, the return-to-work items of the GOSE are not fully aligned with the separate RTW score we used, resulting in patients scoring “favorable” outcome on the GOSE but “unfavorable” on the RTW score.

The results suggest that behavioral disturbances also have an impact on social life and relations. Almost 15% of patients had relational problems and 40% of the relations were ended. This was in line with a study that also showed a comparable number of received supportive care in 8–20% of spouses (34). No significant difference existed between the support received by caregivers in mTBI and mod/sevTBI. The percentages of relational problems and ended relationships we found might even have been higher, as not all patients have reported these problems specifically or have been asked about this at the outpatient visit. On the other hand, relational problems may have existed before sustaining a TBI. Nevertheless, our findings underline the awareness of the impact of behavioral disturbances after TBI and the necessity of long-term care for both patients and caregivers.

When evaluating the complete pathway of care of all TBI patients with behavioral disturbances in this study, almost all patients returned home despite the fact that one in four patients had serious behavioral disturbances. More than half of the patients were participating in a rehabilitation program within the first six months after injury. A favorable outcome was found in 54% of patients within a rehabilitation program compared to 47% in patients without active rehabilitation; with RTW in 60 and 61% respectively. Studies regarding this issue are very limited, because previous studies have focused on the pathways of care in all TBI patients and not specifically on TBI patients with behavioral disturbances (22, 35). In our cohort, only a small percentage of the patients (5%) was temporarily admitted to a psychiatric institution. In the subacute phase after injury these patients did not fit criteria for physical and/or cognitive rehabilitation and were (temporarily) admitted to a nursing home. Ultimately, few patients stayed permanently in a nursing home: in total one in ten of patients with mod/sevTBI and only two patients with mTBI, the latter may also have been related to their age (respectively 78 and 88 years old). Compared to a previous study (22), more patients stayed permanently in a nursing home. This finding suggests that patients end up more often in a nursing home in the long term when behavioral disturbances are present, as in the aforementioned study only 67% patients had behavioral impairments.

We also aimed to find associations of different demographic, clinical, and radiological characteristics with behavioral disturbances. Pre-injury mental health problems and substance abuse in mTBI patients were significantly associated with behavioral disturbances, but this was, noticeably, not found in the mod/sevTBI category. It is possible that this last group is so severely impaired that the presence of pre-injury mental health problems/substance abuse is less relevant for definitive outcome in contrast to the traumatic brain injury itself. In contrast to previous studies (17, 18, 36), we did not find any association between structural frontal and/or temporal traumatic brain lesions and the presence of behavioral disturbances or overall outcome. This could be explained by the fact that we only analyzed a preselected patient group with behavioral disturbances and that half of these patients had mild TBI in which no relation is present between localization of lesions and behavioral disturbances. Furthermore, we analyzed associations with behavioral disturbances in general and not with specific behavioral characterizes such as aggression or apathy, which was more common in previous studies (18, 19, 37).

### Study Limitations

Several limitations have to be addressed. First, data were collected and interpreted retrospectively using information from our database and the patient charts which resulted in missing and/or incomplete data. Not always complete information was found on the caregivers. Therefore caregiver burden might have been underrated in this cohort. Outcome has been determined at the final stage of the rehabilitation, which occurs at a different moment depending on injury severity. mTBI patients mostly reached their final stage after 6 months, while mod/sevTBI patients mostly reach this final stage one year after injury. Nonetheless, these time intervals are regarded as appropriate to measure a relative stable outcome in all studies on TBI.

## Conclusions

Our study shows a high prevalence of serious behavioral disturbances in patients with various severities of TBI. Half of the patients were not able to return to work in both severity categories suggesting that the presence of behavioral disturbances and not the mere severity influences work resumption. Almost all patients returned home with impact on social life and caregivers resulting in relational problems and the need of support for one in five caregivers. Our findings warrant further research focusing on the impact of behavioral disturbances on work resumption and social life, and the provision of early and appropriate care including the support for caregivers.

## Data Availability Statement

The datasets generated for this study are available on request to the corresponding author.

## Ethics Statement

The studies involving human participants were reviewed and approved by Medical ethical committee university medical center Groningen. The ethics committee waived the requirement of written informed consent for participation.

## Author Contributions

MT drafted the manuscript and performed the acquisition and analysis of the data. BJ contributed to the analysis and interpretation of data. MS and JS revised the work critically and interpreted the data. JN contributed to the design of the study and interpretation of data. All authors provide approval for publication of the content and agree to be accountable for all aspects of the work in ensuring that questions related to the accuracy or integrity of any part of the work are appropriately investigated and resolved.

### Conflict of Interest

The authors declare that the research was conducted in the absence of any commercial or financial relationships that could be construed as a potential conflict of interest.
